# Multilabel Classification Methods for Human Activity Recognition: A Comparison of Algorithms

**DOI:** 10.3390/s22062353

**Published:** 2022-03-18

**Authors:** Athanasios Lentzas, Eleana Dalagdi, Dimitris Vrakas

**Affiliations:** Faculty of Sciences, School of Informatics, Aristotle University of Thessaloniki, 541 24 Thessaloniki, Greece; edalagdi@csd.auth.gr (E.D.); dvrakas@csd.auth.gr (D.V.)

**Keywords:** activity recognition, multilabel classification, smart home, ambient sensors, ensemble learning

## Abstract

As the world’s population is aging, and since access to ambient sensors has become easier over the past years, activity recognition in smart home installations has gained increased scientific interest. The majority of published papers in the literature focus on single-resident activity recognition. While this is an important area, especially when focusing on elderly people living alone, multi-resident activity recognition has potentially more applications in smart homes. Activity recognition for multiple residents acting concurrently can be treated as a multilabel classification problem (MLC). In this study, an experimental comparison between different MLC algorithms is attempted. Three different techniques were implemented: *RAkEL${}_{d}$*, classifier chains, and binary relevance. These methods are evaluated using the ARAS and CASAS public datasets. Results obtained from experiments have shown that using MLC can recognize activities performed by multiple people with high accuracy. While *RAkEL${}_{d}$* had the best performance, the rest of the methods had on-par results.

## 1. Introduction

Human activity recognition (HAR) is a research area with increased scientific interest. HAR is the task of correctly recognizing human actions and activities. Its applications vary from athlete and patient monitoring [1,2] to elderly care [3,4]. As IoT devices have become more accessible, solutions that use activity recognition are easier to deploy in smart home installations.

The main purpose of an activity recognition system is to correctly recognize human actions and inform any agent interested about those actions. Recognizing performed activities is a process that, in most cases, happens in real time, as the results are used immediately. The majority of published works focus on recognizing the actions of a single individual [5,6]. While recognizing the activity of only one person is important when monitoring elderly people living alone, it is also critical to recognize activities performed by multiple humans operating in the same space. Information about multiple actions and the way humans interact while performing those activities could provide additional insight about the actions’ context.

Machine learning approaches are a common way to recognize actions performed by humans [4,6]. In a HAR system, usually, multiple activities are recognized; thus, recognizing multiple activites is addressed as a multiclass classification problem [7]. Given a sensory input, the trained model assigns one class (i.e., activity) to that specific input. Multiclass classification algorithms have increased accuracy [8] when only a single person is present. When more than one individual is acting, the problem must be reformed as a multilabel classification problem.

Multilabel classification (MLC) is a classification task where multiple labels may be assigned to each input [9]. Instead of selecting precisely one class out of many, as multiclass classification does, MLC returns one or more classes. It is worth mentioning that there is no constraint on how many classes an input can be assigned to.

Treating HAR as a multilabel classification problem will allow activity recognition for residents acting concurrently. Sensory information, containing activity-related data for multiple people acting concurrently, is used as input.

The purpose of this paper is to perform a benchmark on multilabel classification algorithms used on HAR. Implemented classifiers were trained to recognize a broad set of activities using multiple ambient sensors (i.e., pressure, contact, etc.). Section 1 is a brief introduction to HAR and multilabel classification. Related work is briefly presented in Section 2, followed by the methodology used in our work in Section 4. Thereafter, the results obtained are presented and discussed. At the end, conclusions are drawn and future work is discussed.

## 2. Related Work

Human activity recognition in smart house installations with a single resident is a research area with rapid growth. Several machine learning techniques have been employed in order to address the problem [4]. Random forests have been proven an effective solution [10,11]; they provide robust and explainable results and are easy to implement and train. Hidden Markov models (HMMs) are also a common approach [12,13]; HMMs are not only used for activity recognition, but for absence and home visit detection as well [13]. Deep learning models specifically developed for HAR [14,15] have been gaining increased interest over the past years. The popularity of those models is due to their ability to handle sequential data and time series, the main form of data generated by ambient sensors used in smart home installations.

Despite the fact that machine learning algorithms can recognize performed actions with impressive accuracy, their performance is sub-par when more than one people interact in the same environment. Benchmarks on multi-resident activity recognition [16,17] provide a baseline for researchers that want to address multi-resident activity recognition. Thus, state-of-the-art algorithms, such as the hidden Markov model (HMM), recurrent neural networks (RNN), k-nearest neighbors (KNN), conditional random field (CRF), random forests (RF), and feed-forward neural networks (FFNN), were evaluated thoroughly in this study. Additionally, sensor placement was evaluated, as the sensor network is important for activity recognition [17].

In order to tackle the multiple residents concurrent activity recognition problem, ensemble techniques have been proposed in the literature. A factorial hidden Markov model (HMM) that models two different chains (one for each resident) was proposed by [18]. An ensemble method based on hidden Markov models was presented, aiming to address multi-resident activity recognition, by [19]; the proposed technique introduced a mixed-dependency model, dealing with the complexity of multiple residents acting concurrently. Combined-label HMMs were also evaluated in [20], where activity labels and observation labels of different residents were combined, generating a sequence of observations, recognized by a conventional HMM. Additionally, a model where activities are linked at each time step was proposed. Both models, combined-label and linked activities, outperformed the baseline HMM models.

As smart homes are dynamic environments not limited to their residents, the need to detect visitors, occupants, and even pets is crucial [20,21]. Calculating entropy measures and comparing them with the standard deviation, visitors can be detected and identified [22]. This method can help activity recognition systems to focus only on sensor activations that are relevant to the main residents of the house.

Incremental decision trees have also been employed [23]. While the results were promising, authors identified that further work is needed to obtain results that are on par with single-resident activity recognition. Deep learning is also often explored as a technique able to recognize activities performed by multiple residents. Recurrent neural networks (RNNs) were evaluated using not only real-world data, but also synthetic data generated with a generative adversarial network [24]. Multi-task learning, i.e., regarding the activity of each resident as a learning task, was paired with zero-shot learning to recognize previously unseen activities [25].

Multi-label classification, while extensively used on multiple domains, such as text [26], video [27], and image [28] classification, etc., have only recently drawn the attention of researchers to the activity recognition field. A comparative experimental study between two well-known MLC algorithms [29] proved that MLC can address concurrent activity recognition effectively. Interaction between actions performed by different people can be identified by transforming the multi-resident activity recognition problem into an MLC problem. Experiments have shown that random forests, when used as base classifiers on a MLC algorithm, have an impressive performance [30]. An evaluation of multilabel classification was performed by [31]. The authors use frequent item mining, a technique commonly used in data mining, in order to generate frequent itemsets as features for each activity. The generated features were used to evaluate MLC algorithms such as multilabel KNN, labelset, and decision trees.

Treating multi-resident HAR as a multi-label classification problem while transforming it to a multi-class problem was investigated by [32]. Decision trees were used as the base classifier and label combination was employed for the problem transformation. Binary relevance has been proposed in the literature for multilabel HAR [33]. The results were competitive with approaches transforming the problem into a classic classification problem. The classifier chains approach considers activity correlation, an important aspect of activity recognition [34]. This proposed approach was able to find underlying correlations between activities and reduce the classification time.

## 3. Our Contribution

As already discussed in the previous sections, multi-resident human activity recognition is a research area that attracts a lot of attention. Multi-class classification techniques, while they yield good results, are far from providing a robust and reliable solution. On the other hand, multilabel classification approaches seem to better recognize concurrent activities, but they have not been investigated to the same extent as multi-class classification.

In our work, we aim to provide a baseline for future research on multilabel classification in multi-resident activity recognition. This will allow future researchers to compare their results with results obtained from three well known-algorithms (considered state-of-the-art). Two datasets were used, ARAS and CASAS, which are extensively used on multi-resident activity recognition.

Furthermore, our results are compared with multilabel and multi-class algorithms used on the same problem. This is to identify whether or not the experimentally evaluated algorithms can provide similar or better results. Lastly, $RAkEL_{d}$ has never been evaluated on a multi-resident activity recognition problem before (based on our search); thus, our work reports results not available in the literature.

## 4. Methodology

MLC can help address the problem of multi-resident activity recognition, as the trained model is able to learn from a diverse set of sensors and assign each example to multiple classes. In our work, several state-of-the-art ($RAkEL_{d}$, classifier chain, binary relevance) MLC algorithms are evaluated. Algorithms were chosen based on previous benchmarks [29,35]. All algorithms were implemented using Python and Scikit-Learn [36]. The features used for training were the values of each sensor, recorded every second. No sliding windows were applied on the datasets.

### 4.1. RAkEL_d_

The random k-labelsets algorithm ($RAkEL_{d}$) [37] is an ensemble method for multilabel classification. Each member of the ensemble is constructed by considering a relatively small, random subset of labels. A single label classifier is trained for the prediction of each element in the powerset of the subset. The benefit of applying single label classifiers to sub-tasks with a feasible number of labels is that label correlation is taken into account.

Constructing the labelsets can be realized by using two different methods: disjoint and overlapping labelsets. In our work, disjoint labelsets were constructed. The label space was partitioned to equal partitions of size *k*. For each partition, a label powerset classifier was trained, and the prediction was the sum of all trained classifiers. Labelset size *k* was set to 3, as, according to authors, it allows the best results. An extensive evaluation was performed on the labelset size [37], and results showed that a smaller number affects the performance of the algorithm positively, with 3 being the value that leads to the best results on multiple datasets. In our work, the labelset was evaluated with different values (*k* = 2,3,4,5) and the value the authors proposed (*k* = 3) was the optimal.

Two different classifiers were used during experimentation: multilayer perceptron (MLP) and random forests. In order to fine tune the parameters of the two used classifiers, different techniques were used. The random forest classifier was tuned using randomized parameter optimization [38]. For every hyperparameter that required tuning, a set or range of values was given. A random search was then performed on these distributions *M* times. A model was trained for each combination and the hyperparameters for the best model were returned.

MLP’s hyperparameters were chosen using quantum genetic algorithms (QGA) [39]. Similarly to randomized parameter optimization, a range or set of values was given. The search space was then explored using QGA. Each set of parameters was represented as a chromosome consisting of qudits. A model was trained for each set of parameters. The evaluation was based on the accuracy of the trained model. Non-optimal chromosomes had a chance to re-initialize instead of converging to the best solution. This method allows for a fast convergence to the best solution while performing a random search, thereby avoiding the local optimal.

### 4.2. Binary Relevance

Binary relevance [40] attempts to transform a multilabel classification problem with *L* labels into *L* binary classification problems. The same classifier is used on all sub problems (random forests in our experiments). The final prediction of binary relevance is the union of all individual label classifiers. Binary relevance is a rather simple algorithm that yields promising results. The simplicity of the technique allows for low modeling complexity, linear to the number of class labels in the label space.

### 4.3. Classifier Chain

Another widely used MLC ensemble method is the classifier chain method [41]. A classifier chain constructs a chain of binary classifiers. The number of classifiers constructed is the same as the number of labels in the dataset. As the classifiers are linked in a chain format, a single classifier can utilize the prediction of all previously trained models. Therefore, a classifier chain reconstructs the multilabel problem into a a multi-class problem, where each label combination is a different class and the number of classifiers is equal to the total number of classes. In our experiments, the binary classifier chosen was random forests, tuned with randomized parameter optimization.

### 4.4. ARAS Dataset

The first dataset used in our work is the ARAS (activity recognition with ambient sensing) human activity recognition dataset [42]. The ARAS dataset contains real-life data from two houses with multiple residents. More specifically, 2 young males (25) were living in house A, and a married couple (35) was living in house B. Several ambient sensors were employed to gather data. Force and pressure sensors were placed under the bed and couches, photocells were placed in the drawers, wardrobes, and refrigerator, contact sensors were placed on the door frames, shower cabin, and cupboards, proximity sensors were placed on the chairs, closet, and taps, sonars were placed on the walls and door frames, temperature sensors were installed near the kitchen and oven, and infrared sensors were placed near the TV. The sensors and the actions associated with them are summarized in Table 1. As a result, 27 different activities were recognized (Table 2).

Residents did not follow a specific activity plan during data acquisition, but continued to behave naturally. The only interaction with the system was battery replacement of the sensors and labeling the ground truth manually. Sensors were sampled at 10 Hz (the IR sensor was sampled at 100 Hz). Sensor events were logged every second, resulting in 86,400 data points for each day.

### 4.5. CASAS Dataset

The CASAS dataset contains data from different participants acting as residents of the same house. Each resident performed 15 unique activities (Table 3). Some activities were performed individually (ind.) while some of them required the cooperation of the participants (co-op). Motion sensors were placed on the ceiling, monitoring movement around the room, and contact switches were placed on objects, registering use events. The apartment was always occupied by two participants and 40 volunteers were recruited.

### 4.6. Data Preprocessing

Before training the classifiers mentioned, the data were preprocessed. The main purpose was to transform the dataset into a format that could be used to train the MLC models. As the encoding of the ARAS dataset labels is sequential and common for all the participants (i.e., sleeping is encoded as 11, washing dishes as 9, etc.), a transformation was applied to distinguish actions performed by different persons. Activities performed by the second individual were re-labeled with sequential numbers by adding 27 to the already existing value. As a result, a “new” set of 27 activities was introduce andd applied only to the second person, while the initial set was only referenced by the first participant.

**Figure 1:**
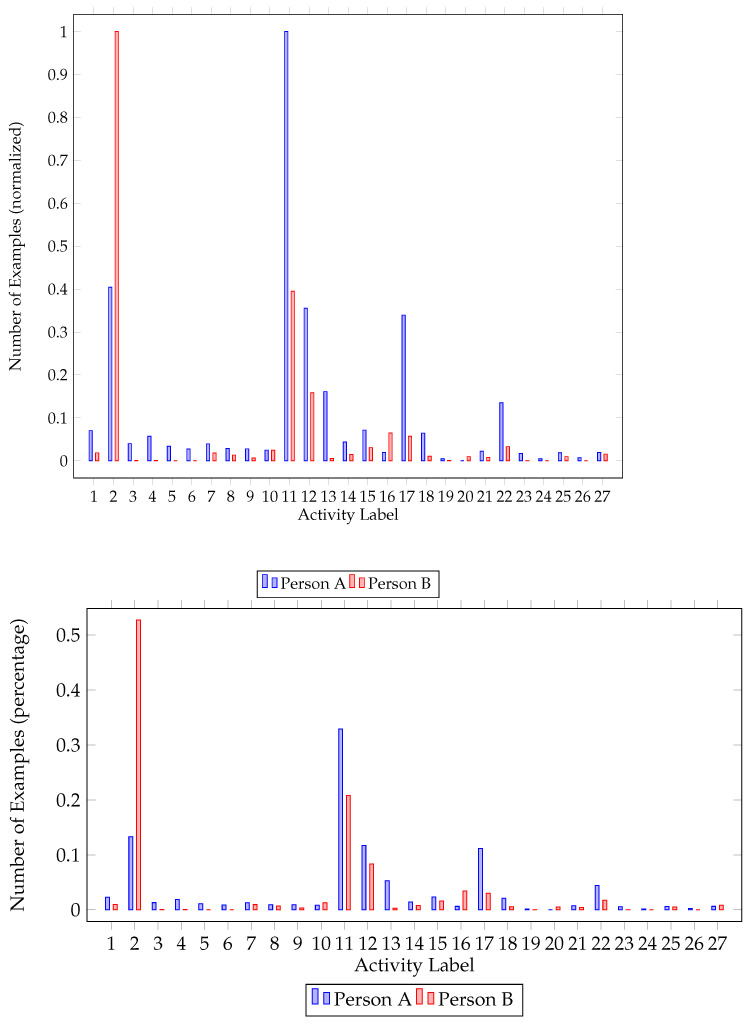
House A class imbalance. Number of classes per resident (normalized) and percentage of each class for the first house.

**Figure 2:**
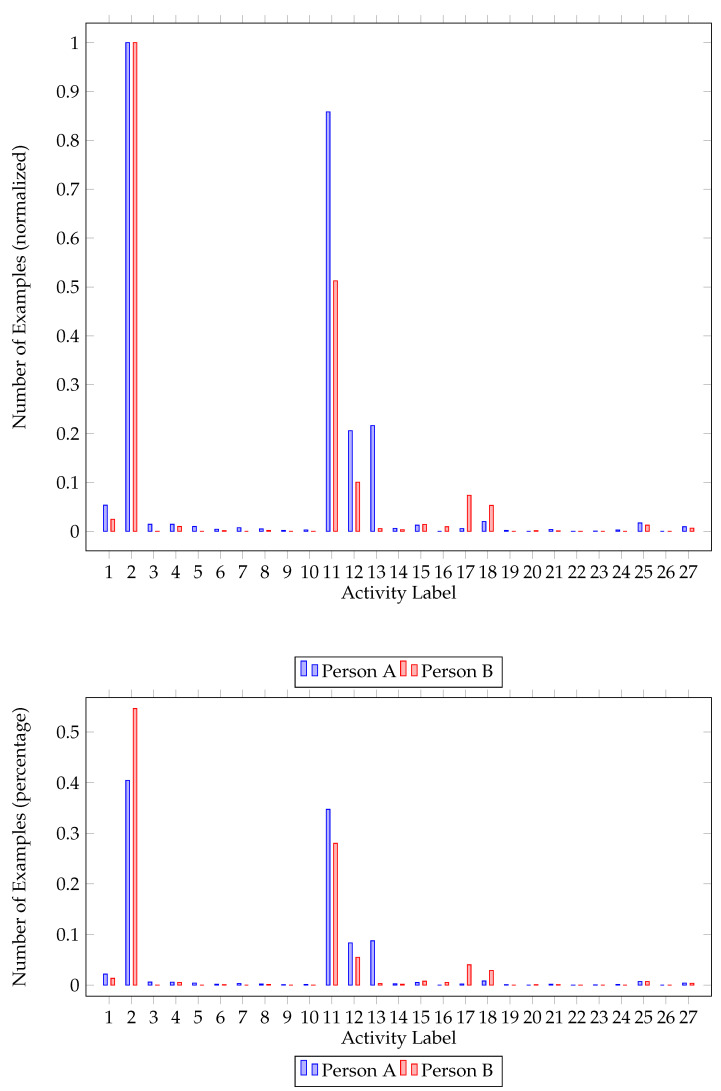
House B class imbalance. Number of classes per resident (normalized) and percentage of each class for the first house.

Class imbalance was also a problem we had to address while preprocessing the dataset. As activities do not occur with the same frequency, i.e., some activities are performed more times than others, the dataset was imbalanced. As seen in Figure 1 and Figure 2, there is a big difference between the major and the minor class in each house. In order to balance the dataset, we upscaled minority classes. To augment our data, the MLSMOTE [43] algorithm (multilabel synthetic minority over-sampling technique), an extension of the SMOTE [44] (synthetic minority over-sampling technique) focused on multilabel datasets, was employed.

Further inspecting class imbalance figures (Figure 1 and Figure 2), one can identify the major and minor classes. In both houses, activities 2 (Going out) and 11 (Sleeping) are the activities mostly performed, followed by watching TV (12) and studying (13). One of the residents in both houses had a major class (going out—2) that represents approximately 55% of the dataset. It is worth mentioning that not all classes are present for all participants. While upscaling the minority classes, activities never recorded were ignored.

As the CASAS dataset has sensor names and values recorded as strings, e.g., ”MD2” as the sensor ID and ”ON” as the sensor event, the data had to be transformed to a more appropriate form. Following the same approach as [45], each sensor was assigned a numerical ID, while values were converted to ”1” or ”0”, based on whether the sensor was triggered or not.

### 4.7. Performance Metrics

Two different metrics were used for evaluation: F1 score and Hamming loss. Before defining the metrics, the type of errors and predictions a classifier can make must be defined:

- False Positives (*FP*): The classifiers predicts a label that is not correct;
- False Negatives (*FN*): The classifier misses a label that exists in the example;
- True Positives (*TP*): The classifier correctly predicts the existence of a label;
- True Negatives (*TN*): The classifier correctly predicts the non-existence of a label.

Using the above information, we can calculate the metrics needed. F1 score is the harmonic mean of Precision (proportion of correct predictions among all predictions) and Recall (proportion of examples of a certain class predicted as members of the class). Equations for each metric can be seen in Equation (1).

(1) $$\begin{array}{ccccc} \mathrm{Precision}=\frac{\sum TP}{\sum TP+\sum FP} & \; & \mathrm{Recall}=\frac{\sum TP}{\sum TP+\sum FN} & \; & F1=2\frac{\mathrm{Precision}*\mathrm{Recall}}{\mathrm{Precision}+\mathrm{Recall}} \end{array}$$

Hamming loss is the fraction of labels that are incorrectly predicted [9]. While on multi-class classification, the Hamming loss is the Hamming distance between true and predicted labels, in MLC, the Hamming label penalizes only the individual classes. The Hamming loss is calculated using Equation (2), where *N* is the total number of data samples, *L* is the total number of available classes, $y_{i,j}$ is the target, and $z_{i,j}$ is the prediction. The xor operator (⊕) returns zero when the target and prediction are the same, and one otherwise. Since the metric is a loss function, the optimal value is zero and the upper bound is one.

(2) $$\mathrm{Hamming}=\frac{1}{\left|N|\times |L\right|}\sum_{i=1}^{N}\sum_{j=1}^{L}⊕\left(y_{i,j},z_{i,j}\right)$$

## 5. Results

The experiments carried out and results are presented in this section. The evaluation was based on 10-fold cross validation. Using the 10-fold cross validation technique is a common practice when evaluating the performance of machine learning models. This technique has numerous advantages, such as reduced bias and variance, etc. The dataset was partitioned into 10 subsets. During each epoch, one subset was used for validation, while the rest (9) were used as training data. The Hamming loss and F1 score were logged after each training epoch.

### 5.1. ARAS Dataset

All proposed models were trained using a whole month’s data. Similar attempts to address the problem trained a different model for each day and averaged the individual results [29]. The average F1 score and Hamming loss for each classifier and house can be seen in Table 4 and Table 5, respectively. Observing the results, one can see that $RAkEL_{d}$ with MLP classifier had the best F1 score and Hamming loss for both houses. Although $RAkEL_{d}$ had a better performance and lower Hamming loss, the difference from the rest of the classifiers was not significant.

As the activity recognition problem is, by its nature, unbalanced, experiments were also performed before applying oversampling. The results, as seen in Table 6 and Table 7, do not differ a lot compared to a balanced dataset. Therefore, multi-label classification can provide robust results on the activity recognition problem, even when the number of examples differs a lot.

An important observation is the significant difference between the results of the two houses. Classifiers evaluated on the second house yielded better results. This observation is justified by the difference in activities performed. Residents of the second house performed less distinct activities, compared to residents of the first house (Figure 1 and Figure 2). This is important, as classification performance is related to the number of activities performed.

**Figure 3:**
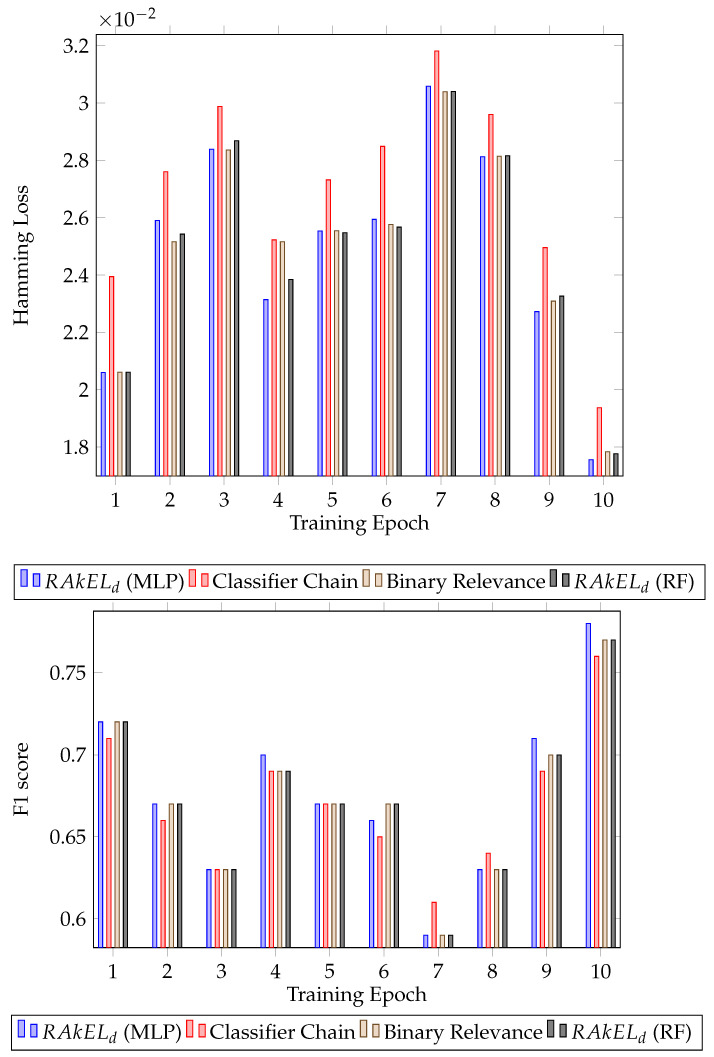
Classification results for House A. Hamming loss for each classifier per validation epoch and F1 score for each classifier per validation epoch.

**Figure 4:**
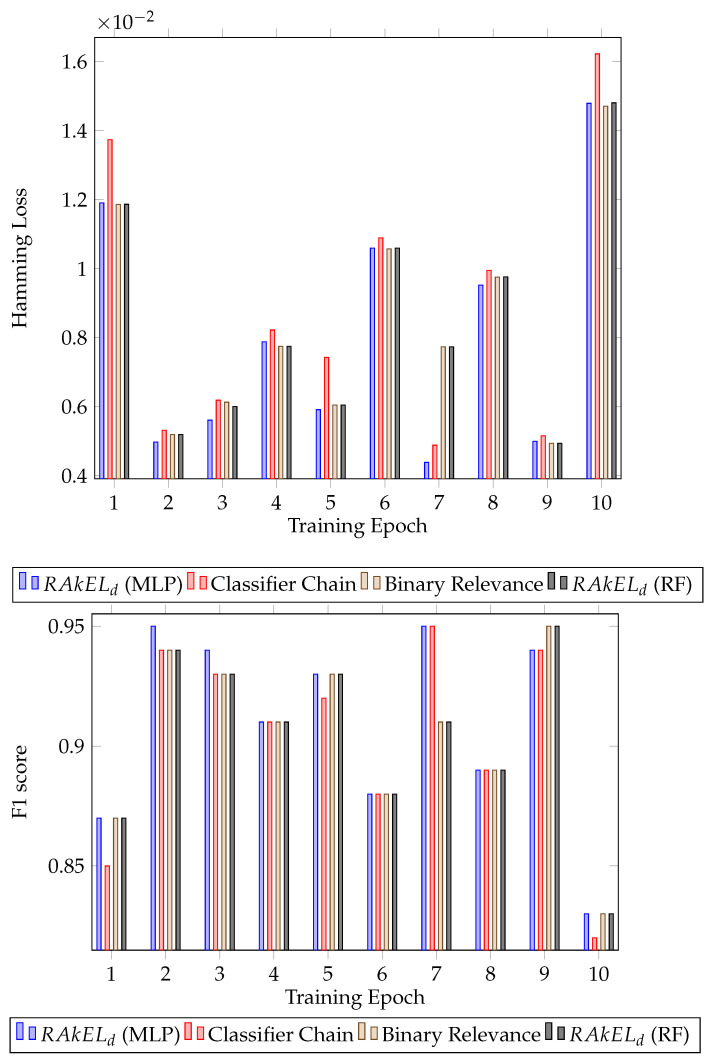
Classification results for House B. Hamming loss for each classifier per validation epoch and F1 score for each classifier per validation epoch.

Further analysing the results, the standard deviation of the F1 score was calculated for each algorithm. For both houses, $RAkEL_{d}$ had the lower standard deviation, 0.041 for the first house ($RAkEL_{d}$ with MLP) and 0.034 for the second house ($RAkEL_{d}$ with random forest). The results for each epoch for all the classifiers can be seen in Figure 3 for the first house and Figure 4 for the second house.

Performing the same experiments on a daily basis, instead of using the data from a whole month, provided the results seen in Table 8 and Table 9. $RAkEL_{d}$, with both classifiers evaluated (MLP and RF), had the best overall performance. The results were consistent and no major deviations were observed between different days. Furthermore, the results of $RAkEL_{d}$ using MLP are compared with the results obtained on the same dataset by [29] (Table 10). The comparison is based on the only common metric, Hamming loss. By comparing the results, one can identify that $RAkEL_{d}$ performed better for both houses.

Analyzing the results of the experiments, one can identify activities that are more prone to misclassification. For House A, the hardest activities to recognize were having lunch and having conversation for residents 1 and 2, respectively. reparing dinner was the least recognized activity for resident 1 in House B and having conversation was least recognized for resident 2. It is worth mentioning that, except for resident 1 (House A), the rest of the least recognized classes were under-sampled. As a result, there were training epochs where the class was not represented in the training data.

### 5.2. CASAS Dataset

Experiments on the CASAS dataset were performed similarly to the ARAS dataset. The same classifiers, binary relevance, classifier chain, *RAkEL${}_{d}$* with random forests, and *RAkEL${}_{d}$* with MLP were used. The evaluation of the CASAS dataset was based on 10-fold cross validation in order to reduce bias and variance. Using the whole CASAS dataset, i.e., 26 days, provided the results seen in Table 11. *RAkEL${}_{d}$* with MLP classifier had the best overall performance when the classifiers were trained and evaluated using the entire dataset. The rest of the classifiers were on par regarding performance, making them a viable solution for multi-resident activity recognition. Similarly to the ARAS dataset experiments, each classifier was evaluated using daily data (Table 12). *RAkEL${}_{d}$* had the best performance with, results the being consistent with the ARAS dataset.

### 5.3. Multilabel and Multi-Class Comparison

Multi-resident activity recognition, as already discussed in Section 2, can be addressed both as a multilabel and multi-class classification problem. In order to further support our position that MLC can be used on multi-resident activity recognition without sacrificing accuracy, a comparison between the two approaches is needed. We did not run any experiments with multi-class models, but we compared our results with results already published in the literature [16].

As seen in Table 13, multilabel classification outperformed multi-class classification. The average F1 score was better for MLC approaches with both datasets. The multi-class classifiers with the best overall performance was recurrent neural networks for the CASAS dataset, and random forests for ARAS. It is worth mentioning that on the ARAS dataset, for House A, there was a significant difference between multilabel and multi-class classification. This is important, as the aforementioned house had increased difficulty, mainly because of the diverse set of activities performed.

## 6. Conclusions

In this work, an experimental evaluation of *RAkEL${}_{d}$*, classifier chain, and binary relevance methods for multi-resident human activity recognition was performed. Experiments were performed on the ARAS and CASAS datasets, two activity recognition datasets with two residents acting concurrently. *RAkEL${}_{d}$* had the best overall performance on both datasets, with the rest of the evaluated classifiers yielding on-par results. When a multi-layered perceptron was used as a base classifier on *RAkEL${}_{d}$*, the performance of the algorithm was improved.

The ARAS dataset was analyzed and the findings showed that it suffers from the class imbalance problem. In order to address this, the minority classes were upscaled before training the classifiers. Balancing the dataset was an important step during data preprocessing, as the major classes represented approximately 90% of the whole data for both houses. Experiments were performed on both a balanced and unbalanced dataset, without any significant difference between the results.

Observing the activity distribution on the dataset, it was observed that residents follow a different activity pattern. Although the dataset has records for 27 different activities, not all of them are present in each house. As house B residents performed less distinct activities, the trained models had a better overall performance.

Results were also compared with multi-class classification methods used on the same set of data, as well as multilabel classification methods already published in the literature. The comparison showed that *RAkEL${}_{d}$*s performance is on par with, if not better than, different multilabel classification approaches, outperforming multi-class classification techniques. This can provide a baseline for future comparisons of multilabel classification solutions used on the multi-resident activity recognition problem.

Future work will be focused on identifying correlations between different activities in multi-resident environments. Some activities could potentially be mutually exclusive (i.e., both residents toileting), or inclusive (i.e., both having a conversation, lunch, etc.). That relationship could be identified and thereby further enhance the performance of classification. As the datasets used in our work do not contain mutually exclusive activities, this aspect is not investigated in this work.

Furthermore, more MLC algorithms should be evaluated. While this study extends already published works on multi-resident activity recognition, there are a lot of techniques that have not yet been evaluated, such as ML-KNN [46], CLARE decision trees [47], and deep neural networks adapted for MLC problems. While our work is tested on two different datasets, proving the robustness of the evaluated algorithms, there are more methods available in the literature. Additionally, hierarchical multilabel classification should be explored. As some activities are correlated in a hierarchical order, e.g., preparing dinner always precedes eating dinner, it is a promising idea to create efficient structures that represent that hierarchical order.

## Figures and Tables

**Table 1:** Sensors and their associated actions.

Sensors	Actions
Force and pressure sensors	Sleeping, sitting, napping
Photo sensor	Opening drawers and wardrobes
Contact sensors	Opening/closing doors, cupboards
Sonar	Presence detection
Temperature sensors	Cooking
Infrared	Watching TV

**Table 2:** Activities available in the ARAS dataset.

Activities		
Going out	Preparing breakfast	Having breakfast
Preparing lunch	Having lunch	Preparing dinner
Having dinner	Washing dishes	Making snack
Sleeping	Watching TV	Studying
Having shower	Toileting	Napping
Using internet	Reading book	Laundry
Shaving	Brushing teeth	Talking on the phone
Listening to music	Cleaning	Having conversation
Changing clothes	Having clothes	Other

**Table 3:** Activities performed by each resident on the CASAS dataset.

Person A	Person B
Filling medication dispenser (ind.)	Hanging up clothes (ind.)
Moving furniture (co-op)	Moving furniture (co-op)
Watering plants (ind.)	Reading magazine (ind.)
Playing checkers (co-op)	Sweeping floor (ind.)
Preparing dinner (ind.)	Playing checkers (co-op)
Reading magazine (ind.)	Setting the table (ind.)
Gathering and packing picnic food (ind.)	Paying bills (co-op)
-	Gathering and packing picnic supplies (co-op)

**Table 4:** Average F1 score per classifier. Inside the parentheses is the confidence interval (96% confidence). The best value can be seen in bold.

Classifier	House A	House A
$RAkEL_{d}$ (MLP)	**0.676** (0.64–0.70)	**0.909** (0.88–0.93)
Classifier chain	0.671 (0.64–0.69)	0.903 (0.87–0.92)
Binary relevance	0.674 (0.64–0.7)	0.904 (0.88–0.92)
$RAkEL_{d}$ (Random forest)	0.674 (0.64–0.7)	0.904 (0.88–0.92)

**Table 5:** Average Hamming loss per classifier (lower is better). Inside the parentheses is the confidence interval (96% confidence). The best value can be seen in bold.

Classifier	House A	House B
$RAkEL_{d}$ (MLP)	0.024846898 (0.022–0.024)	**0.008057439 ** (0.0059–0.01)
Classifier chain	0.026821258 (0.024–0.029)	0.008799636 (0.0065–0.0088)
Binary relevance	**0.02482958** (0.022–0.027)	0.00846863 (0.0065–0.0084)
$RAkEL_{d}$ (Random forest)	0.024929984 (0.022–0.027)	0.008469172 (0.0065–0.0084)

**Table 6:** Average F1 score per classifier trained on an unbalanced dataset. Inside the parentheses is the confidence interval (96% confidence). The best value can be seen in bold.

Classifier	House A	House B
$RAkEL_{d}$ (MLP)	**0.679** (0.64–0.72)	**0.911** (0.89–0.93)
Classifier chain	0.673 (0.64–0.70)	0.905 (0.9–0.93)
Binary relevance	0.675 (0.64–0.70)	0.906 (0.88–0.92)
$RAkEL_{d}$ (Random forest)	0.676 (0.64–0.71)	**0.911** (0.89–0.93)

**Table 7:** Average Hamming loss per classifier (lower is better) trained on an unbalanced dataset. Inside the parentheses is the confidence interval (96% confidence). The best value can be seen in bold.

Classifier	House A	House B
$RAkEL_{d}$ (MLP)	**0.024651636** (0.022–0.028)	**0.007962516** (0.0059–0.01)
Classifier chain	0.026690177 (0.024–0.03)	0.008666588 (0.0065–0.01)
Binary relevance	0.026660177 (0.024–0.03)	0.00837087 (0.0065–0.01)
$RAkEL_{d}$ (Random forest)	0.02475237 (0.022–0.028)	0.008020018 (0.006–0.01)

**Table 8:** Average F1 score and Hamming loss per day for House A. Best value per day can be seen in bold.

Day	${\mathrm{RAkEL}}_{d}$ (MLP)		Classifier Chain		Binary Relevance		${\mathrm{RAkEL}}_{d}$ (Random Forest)	
	F1	Hamming	F1	Hamming	F1	Hamming	F1	Hamming
1	0.808	0.036	0.791	0.0412	0.677	0.0555	**0.81**	**0.0328**
2	**0.86**	**0.033**	0.858	0.0366	0.841	0.0363	0.853	0.0454
3	**0.769**	**0.036**	0.696	0.0498	0.7	0.0495	**0.769**	0.0375
4	0.811	**0.029**	0.716	0.0503	0.789	0.0333	**0.82**	0.0298
5	0.795	0.025	0.732	0.0399	0.723	0.0357	**0.826**	**0.0219**
6	0.807	**0.024**	0.735	0.0373	0.721	0.0372	**0.823**	0.0255
7	0.811	0.036	0.749	0.0445	0.733	0.0475	**0.865**	**0.0253**
8	**0.848**	**0.02**	0.764	0.0327	0.692	0.0457	0.837	0.0216
9	0.782	**0.03**	0.688	0.0471	0.707	0.0386	**0.793**	0.035
10	**0.837**	**0.026**	0.726	0.0426	0.829	0.0258	0.835	0.0269
11	0.786	**0.042**	0.728	0.0507	0.805	0.0435	**0.794**	**0.042**
12	0.798	0.045	0.776	0.044	0.806	0.0451	**0.817**	**0.0378**
13	0.781	0.039	0.763	0.0445	0.796	0.0273	**0.834**	**0.0332**
14	0.791	0.064	0.752	0.0713	0.781	0.0706	**0.82**	**0.0515**
15	0.758	0.068	0.722	0.0645	0.762	0.0687	**0.8**	**0.0516**
16	0.822	0.027	0.73	0.0406	0.787	0.0339	**0.846**	**0.0242**
17	**0.819**	**0.027**	0.711	0.0487	0.814	0.0325	0.806	0.0312
18	**0.826**	**0.033**	0.774	0.0413	0.783	0.0446	0.813	0.0366
19	0.811	0.035	0.733	0.0412	0.798	0.0342	**0.828**	**0.0297**
20	0.786	**0.063**	0.729	0.0707	0.777	0.06	**0.809**	0.066
21	**0.804**	0.047	0.749	0.0573	0.747	0.0441	0.794	**0.0415**
22	0.828	0.039	0.843	0.0409	0.822	0.043	**0.87**	**0.0361**
23	0.78	0.039	0.715	0.0556	0.77	0.0403	**0.786**	**0.0338**
24	0.804	0.033	0.765	0.0371	**0.828**	**0.0262**	0.817	0.0282
25	0.782	0.051	0.708	0.0683	0.773	0.0586	**0.815**	**0.0477**
26	**0.807**	**0.039**	0.648	0.0588	0.755	0.0481	0.795	0.0435
27	0.849	0.03	0.866	0.0246	**0.917**	**0.0191**	0.891	0.0235
28	0.816	0.04	0.733	0.0488	0.82	**0.0341**	**0.833**	0.0385
29	0.838	0.043	0.775	0.0573	**0.862**	**0.0377**	0.852	0.0408
30	0.881	0.028	0.877	0.0295	0.872	0.0298	**0.917**	**0.0226**

**Table 9:** Average F1 score and Hamming loss per day for House B. Best value per day can be seen in bold.

Day	${\mathrm{RAkEL}}_{d}$ (MLP)		Classifier Chain		Binary Relevance		${\mathrm{RAkEL}}_{d}$ (Random Forest)	
	F1	Hamming	F1	Hamming	F1	Hamming	F1	Hamming
1	0.802	0.045	0.783	0.0457	0.774	0.0466	**0.812**	**0.044**
2	0.902	**0.0216**	**0.903**	0.0223	0.881	0.022	0.897	**0.0216**
3	0.958	0.011	0.957	**0.0108**	0.952	0.0113	**0.96**	0.0111
4	**0.858**	**0.0236**	0.843	0.0282	0.825	0.0279	0.852	0.0276
5	**0.91**	**0.0248**	**0.91**	**0.0248**	0.901	0.0249	0.908	0.0255
6	0.934	**0.0134**	0.94	0.0135	0.929	0.0136	**0.941**	**0.0134**
7	**0.903**	**0.0215**	0.902	0.023	0.887	0.0233	0.891	0.0232
8	0.948	**0.0167**	0.948	0.0175	0.939	0.0176	**0.95**	0.0175
9	0.947	0.0107	0.946	0.0121	0.944	**0.0106**	**0.951**	0.0113
10	**0.924**	**0.0145**	0.895	0.0196	0.898	0.0175	0.901	0.0188
11	0.92	**0.0185**	**0.953**	0.0189	0.901	0.0189	0.92	**0.0185**
12	**0.922**	**0.0172**	0.918	0.0175	0.91	0.0173	0.92	**0.0172**
13	**0.96**	**0.0096**	0.937	0.0142	0.951	0.0106	0.956	0.0114
14	0.984	**0.0062**	0.976	0.0074	0.981	0.0065	**0.986**	0.0074
15	0.926	**0.0194**	0.924	0.0207	0.92	0.0202	**0.928**	0.0202
16	0.908	0.0173	**0.919**	**0.0164**	0.899	0.0179	0.912	0.0177
17	**0.887**	0.0226	0.881	0.023	0.87	**0.0219**	0.885	0.0235
18	0.96	**0.0096**	0.954	**0.0096**	0.951	**0.0096**	**0.961**	**0.0096**
19	**0.928**	**0.015**	0.917	0.0169	0.908	0.0166	0.92	0.0165
20	0.951	**0.0166**	**0.982**	0.017	0.946	**0.0166**	0.952	**0.0166**
21	**0.953**	0.0153	0.929	0.0199	0.952	**0.0125**	0.95	0.0157
22	**0.953**	0.0131	**0.953**	**0.0104**	0.941	0.013	0.95	0.0131
23	**0.914**	**0.0256**	0.906	**0.0256**	0.899	**0.0256**	0.913	**0.0256**
24	**0.903**	**0.0174**	0.88	0.022	0.878	0.0186	0.892	0.0194
25	**0.96**	0.0121	0.954	0.0122	0.951	0.0124	0.957	0.0124
26	**0.952**	**0.0126**	0.948	0.0145	0.943	0.0139	0.951	0.0139
27	**0.9**	0.0214	0.888	0.0231	**0.9**	0.0208	0.897	0.0216
28	0.847	0.0262	0.83	0.031	0.846	**0.0254**	**0.86**	**0.0245**
29	0.932	**0.0209**	0.93	0.0217	**0.933**	0.0205	0.929	0.0235
30	0.918	0.0169	0.905	0.0204	0.914	**0.0163**	**0.925**	**0.0163**

**Table 10:** Hamming loss comparison between $RAkEL_{d}$ (MLP) and results presented in [29]. Best value per day can be seen in bold.

Day	House A		House B	
	$RAkEL_{d}$ (MLP)	[29]	$RAkEL_{d}$ (MLP)	[29]
1	**0.036**	0.107	**0.045**	0.058
2	**0.033**	0.083	**0.0216**	0.043
3	**0.036**	0.09	**0.011**	0.023
4	**0.029**	0.119	**0.0236**	0.039
5	**0.025**	0.123	**0.0248**	0.031
6	**0.024**	0.183	0.0134	**0.009**
7	**0.036**	0.116	**0.0215**	0.018
8	**0.02**	0.104	**0.0167**	0.02
9	**0.03**	0.192	**0.0107**	0.014
10	**0.026**	0.112	**0.0145**	0.023
11	**0.042**	0.105	**0.0185**	0.049
12	**0.045**	0.101	**0.0172**	0.021
13	**0.039**	0.077	**0.0096**	0.011
14	**0.064**	0.107	0.0062	**0.006**
15	**0.068**	0.158	**0.0194**	0.044
16	**0.027**	0.149	**0.0173**	0.054
17	**0.027**	0.083	**0.0226**	0.051
18	**0.033**	0.102	0.0096	**0.008**
19	**0.035**	0.164	**0.015**	0.022
20	**0.063**	0.134	0.0166	**0.014**
21	**0.047**	0.207	**0.0153**	0.027
22	**0.039**	0.145	**0.0131**	0.028
23	**0.039**	0.182	**0.0256**	0.039
24	**0.033**	0.095	**0.0174**	0.026
25	**0.051**	0.124	**0.0121**	0.018
26	**0.039**	0.12	**0.0126**	0.015
27	**0.03**	0.086	0.0214	**0.018**
28	**0.04**	0.096	**0.0262**	0.064
29	**0.043**	0.079	**0.0209**	0.025
30	**0.028**	0.05	**0.0169**	0.041

**Table 11:** Average F1 score & Hamming loss per classifier (CASAS dataset). Inside the parentheses is the confidence interval (96% confidence). Best values can be seen in bold.

Classifier	F1	Hamming Loss
$RAkEL_{d}$ (MLP)	**0.912** (0.89–0.92)	**0.0080326** (0.0072–0.01)
Classifier chain	0.887 (0.87–0.9)	0.009235 (0.0075–0.012
Binary relevance	0.89 (0.88–0.91)	0.0089681 (0.0073–0.011)
$RAkEL_{d}$ (Random forest)	0.9 (0.9–0.91)	0.008220018 (0.0058–0.01

**Table 12:** Average F1 score and Hamming loss per day for the CASAS Dataset. Best values per day can be seen in bold.

Day	${\mathrm{RAkEL}}_{d}$ (MLP)		Classifier Chain		Binary Relevance		${\mathrm{RAkEL}}_{d}$ (Random Forest)	
	F1	Hamming	F1	Hamming	F1	Hamming	F1	Hamming
1	0.86	0.033	0.732	0.0399	0.793	0.0355	**0.864**	**0.0301**
2	**0.886**	**0.0246**	0.826	0.035	0.768	0.0324	0.875	0.0296
3	0.7925	**0.0285**	0.798	0.047	0.782	0.0373	**0.925**	0.0338
4	**0.806**	**0.0214**	0.759	0.0314	0.798	0.0257	0.805	0.0337
5	0.827	0.034	0.762	0.0364	0.792	0.0269	**0.863**	**0.0211**
6	0.809	0.0264	0.763	0.0391	0.768	0.035	**0.89**	**0.0249**
7	0.787	0.025	0.749	0.0298	0.744	0.0367	**0.866**	**0.0154**
8	**0.942**	**0.012**	0.776	0.0299	0.847	0.0259	0.875	0.0155
9	0.828	0.034	0.856	0.0375	0.73	**0.0315**	**0.877**	**0.0315**
10	**0.926**	0.0205	0.841	0.025	0.805	0.0257	0.872	**0.014**
11	0.852	0.0223	0.731	0.0304	0.721	0.0303	**0.871**	**0.0189**
12	0.823	**0.0119**	0.797	0.034	0.833	0.0331	**0.902**	0.0295
13	**0.917**	**0.0213**	0.828	0.0223	0.797	0.0343	0.911	0.0285
14	**0.925**	0.022	0.868	0.0235	0.728	0.0375	0.844	**0.015**
15	0.854	**0.0225**	0.808	0.0303	0.787	0.0385	**0.909**	0.0306
16	0.902	**0.025**	0.755	0.0384	0.88	0.0221	**0.911**	0.03
17	**0.97**	**0.013**	0.781	0.0362	0.799	0.0258	0.869	0.0328
18	**0.916**	**0.023**	0.893	0.0269	0.899	0.0345	0.871	0.026
19	**0.929**	**0.016**	0.84	0.0287	0.78	0.0241	0.835	0.0201
20	0.889	0.023	0.815	0.0302	0.73	0.0224	**0.904**	**0.0152**
21	0.866	0.0345	0.823	0.025	0.856	0.0222	**0.89**	**0.0107**
22	**0.93**	**0.0103**	0.855	0.0378	0.735	0.035	0.899	0.027
23	**0.89**	**0.0226**	0.887	0.0295	0.81	0.0275	0.887	0.0324
24	**0.914**	**0.0218**	0.853	0.035	0.796	0.022	0.867	0.0227
25	0.858	0.0392	0.79	0.0361	0.8	0.0371	**0.863**	**0.0232**
26	0.847	0.0563	0.727	0.0345	0.823	**0.0203**	**0.909**	0.0336

**Table 13:** F1 score comparison between multilabel and multi-class classification approaches. Best values are in bold.

Method	ARAS House A	ARAS House B	CASAS
Multilabel	**0.679**	**0.911**	**0.902**
Multi-class [16]	0.5987	0.8796	0.8227

## Data Availability

ARAS and CASAS datasets are publicly available. ARAS dataset can be found at www.cmpe.boun.edu.tr/aras/ (accessed on 14 January 2022) and CASAS dataset at casas.wsu.edu/datasets/ (accessed on 14 January 2022). For experiment setup you can contact the corresponding author.

## Abbreviations

The following abbreviations are used in this manuscript:

HAR	Human activity recognition
HMM	Hidden Markov model
KNN	K-nearest neighbour
RNN	Recurrent neural network
RF	Random forests
FNN	Feed-forward neural network
MLC	Multilabel classification
MLSMOTE	Multilabel synthetic minority over-sampling technique
SMOTE	Synthetic minority over-sampling technique
$RAkEL_{d}$	Random k-labelsets multilabel classifier
MLP	Multilayer perceptron
QGA	Quantum genetic algorithms
